# Insights from bioinformatics analysis reveal that lipopolysaccharide induces activation of chemokine-related signaling pathways in human nasal epithelial cells

**DOI:** 10.1038/s41598-024-58317-y

**Published:** 2024-04-01

**Authors:** Shaolin Tan, Yuelong Gu, Ying Zhu, Chunyu Luo, Zhipeng Li, Hai Lin, Weitian Zhang

**Affiliations:** 1 Postgraduate Training Base of Shanghai Sixth People’s Hospital, Jinzhou Medical University, Shanghai, China; 2 Department of Otolaryngology-Head and Neck Surgery & Allergy Center, Shanghai Sixth People’s Hospital Affiliated to Shanghai Jiao Tong University School of Medicine, Shanghai, China; 3 Otolaryngological Institute, Shanghai Jiao Tong University, Shanghai, China; 4 Shanghai Key Laboratory of Sleep Disordered Breathing, Shanghai, China

**Keywords:** Computational biology and bioinformatics, Functional clustering, Gene ontology, High-throughput screening, Sequence annotation

## Abstract

Lipopolysaccharide (LPS) is known to elicit a robust immune response. This study aimed to investigate the impact of LPS on the transcriptome of human nasal epithelial cells (HNEpC). HNEpC were cultured and stimulated with LPS (1 μg/mL) or an equivalent amount of normal culture medium. Subsequently, total RNA was extracted, purified, and sequenced using next-generation RNA sequencing technology. Differentially expressed genes (DEGs) were identified and subjected to functional enrichment analysis. A protein–protein interaction (PPI) network of DEGs was constructed, followed by Ingenuity Pathway Analysis (IPA) to identify molecular pathways influenced by LPS exposure on HNEpC. Validation of key genes was performed using quantitative real-time PCR (qRT-PCR). A total of 97 DEGs, comprising 48 up-regulated genes and 49 down-regulated genes, were identified. Results from functional enrichment analysis, PPI, and IPA indicated that DEGs were predominantly enriched in chemokine-related signaling pathways. Subsequent qRT-PCR validation demonstrated significant upregulation of key genes in these pathways in LPS-treated HNEpC compared to control cells. In conclusion, LPS intervention profoundly altered the transcriptome of HNEpC, potentially exacerbating inflammatory responses through the activation of chemokine-related signaling pathways.

## Introduction

Lipopolysaccharide (LPS), also known as endotoxin, is a complex molecule composed of lipids and polysaccharides (glycolipids), constituting a major component of the outer membrane in certain bacteria, particularly gram-negative bacteria^1–3^. LPS exhibits intricate biological activities, serving as an immune enhancer that elicits a robust immune response during bacterial infections. It activates various immune cells, including T lymphocytes, B lymphocytes, and macrophages, while also promoting cytokine production, complement activation, and overall immune enhancement^4–6^.

Nasal epithelial cells, forming the outermost barrier of nasal mucosal tissue, are the primary targets of external pathogenic agents such as bacteria, viruses, and environmental pollutants. This epithelial barrier plays a crucial role in preventing the entry of harmful substances and microorganisms into the lower respiratory tracts, including the bronchi and lungs^7,8^. Numerous studies have demonstrated that external bacteria can invade nasal epithelial cells, compromising the epithelial barrier and triggering a cascade of inflammatory and immune responses^9,10^. LPS is commonly employed to mimic external pathogenic bacteria in research models studying the invasion of nasal epithelial cells^11^.

As described above, LPS plays a key role in the inflammatory response associated with respiratory diseases. Nasal epithelial cells serve as the primary target for external pathogenic bacteria. However, the precise involvement of LPS in provoking inflammatory responses in nasal epithelial cells, its impact on their transcriptome, and the specific underlying mechanisms remain uncertain. In light of these uncertainties, our aim was to investigate the influence of LPS on the transcriptome of human nasal epithelial cells (HNEpC) in vitro. Our investigation encompassed the identification of differentially expressed genes (DEGs), the execution of principal component analysis (PCA), and the undertaking of comprehensive functional analyses, which included gene ontology (GO), Kyoto Encyclopedia of Genes and Genomes (KEGG), and Gene Set Enrichment Analysis (GSEA). Additionally, we conducted Protein–protein interaction (PPI) network analysis and employed Ingenuity Pathways Analysis (IPA). The overarching objective was to elucidate the specific mechanisms governing the inflammatory responses induced by LPS in HNEpC.

## Materials and methods

### HNEpC culture and LPS stimulation

HNEpC cells (PromoCell, C-12620, Heidelberg, Germany) were cultured in airway epithelial cell basal medium (PromoCell, C-21060) supplemented with the growth medium kit (PromoCell, C-21160) in a 5% CO_2_ incubator at 37 °C. Subsequently, HNEpC cells were cultured and stimulated with LPS (1 μg/mL, L2880, Sigma-Aldrich, St. Louis, MO, USA) or an equal volume of normal culture medium for 6 h. All concentrations of stimulators mentioned herein represent the final concentrations.

### Cell viability tests

HNEpC cells were diluted to 1 × 10^5^/mL with airway epithelial cell culture medium and seeded in sterile 96-well culture plates in a 5% CO_2_ incubator at 37 °C for 24 h. The LPS stock solution was diluted to various concentrations (0.125, 0.25, 0.5, 1, 2, and 4 μg/mL) with airway epithelial cell culture medium. Control wells received an equal volume of culture medium, and blank wells had no cells. The MTT Assay Kit was employed to determine the number of viable cells using the formula: Cell viability = (LPS-treated well − blank well)/(control well − blank well) × 100%. Additional details are available in the Supporting Information of this article.

### Transcriptome sequencing of HNEpC stimulated by LPS

Transcriptome sequencing of HNEpC cells stimulated by LPS was conducted for further bioinformatics analysis. Initially, HNEpC cells were cultured in a 6-well plate at 37 °C with 5% CO_2_. Before stimulation, the LPS stock solution was diluted to 1 μg/mL with normal culture medium. Three experimental groups of HNEpC cells were exposed to 2 mL of 1 μg/mL LPS suspension for 6 h, and three control groups were treated with the same amount of normal culture medium for the same duration. Subsequently, total RNA of HNEpC cells was extracted, sent to Shanghai Bohao Biotechnology Co., Ltd. (Shanghai, China), and subjected to RNA extraction, purification, RNA-seq, data processing, and bioinformatics analysis. The statistical summary of the HNEpC transcriptome for sequencing is presented in Table S1, and raw expression data were deposited in the Gene Expression Omnibus (GEO) database (GSE241554).

### PCA, DEGs identification, GO, KEGG and GSEA analyses in our transcriptome data

PCA, DEGs identification, GO, KEGG, and GSEA analyses on our transcriptome data were performed using the "ggplot2," "edgeR," and "clusterProfiler" R packages. The "edgeR" R package was applied to assess the expression levels between LPS-treated groups and control groups, with the criteria for DEG selection being adjusted *p* value (*q* value) < 0.05 and |Log2 fold change (Log2FC)|> 1. GO, KEGG, and GSEA analyses utilized the "clusterProfiler" R package, considering pathways with a *q* value < 0.05 as significantly enriched. The gene list was pre-ranked by fold changes for GSEA, using canonical pathways gene sets from the KEGG pathway database (mSigDB C2 category) downloaded from the "Molecular Signatures Database v2023.1." Normalized enrichment score (NES) was used to evaluate enrichment degree, with a significance threshold of *p* < 0.05, false discovery rate (FDR) < 0.25, and |NES|> 1 deemed statistically significant.

### PPI network analysis and construction

PPI network analysis was conducted using the Search Tool for the Retrieval of Interacting Genes (STRING) (version 11.5, https://string-db.org) with a threshold of combined score > 0.4, subsequently visualized by Cytoscape (version: 3.9.1, https://cytoscape.org/)^12^. The closeness parameter of each node was calculated using the "cytoHubba" plugin of Cytoscape and used to rank all nodes.

### IPA analysis

The results of DEGs containing gene identifiers, corresponding log2 fold changes, and FDR values were uploaded into the IPA software (version 1.0, https://digitalinsights.qiagen.com/; Qiagen Bioinformatics, Redwood City, CA, USA) and analyzed with the "core analysis" function. Enrichment pathways of DEGs were generated based on the Ingenuity Pathway Knowledge Data Base. The results of enriched pathways were ranked by –log10 (*p*-value).

### Quantitative real-time PCR (qRT-PCR) verification

To verify the results of RNA sequencing, key DEGs including CXCL1, CXCL8, CXCL10, CCL20, RELB, LCN2, BIRC3, NFKBIZ, IRAK2, PI3, SAA1, CSF3, and CSF1 were chosen for qRT-PCR (Table S5) assay on the RNA of HNEpC. qRT-PCR was performed using the SYBR Green method with specific primers (Table S6). Additional information is available in the Supporting Information of this article.

In addition, in order to further verify the results of transcriptome sequencing and bioinformatics analysis, we isolated primary human nasal epithelial cells (pHNEpC) from inferior turbinate tissues obtained from six patients (three males and three females) of median age 38 years (range 19 to 58 years) undergoing septoplasty for a deviated septum. These pHNEpC were seeded in 6-well culture plates coated with collagen (5005, PureCol® Type I Collagen Solution, Advanced BioMatrix) and cultured in serum-free bronchial epithelial cell growth medium (BEGM, Lonza, Walkersville, Md) in an incubator at 37 °C and 5% CO_2_. Then, pHNEpC cells were cultured and stimulated with LPS (1 μg/mL) or an equal volume of normal culture medium for 6 h. Following stimulation, total RNA of these cells was extracted for qRT-PCR assay.

This study was approved by the Ethical Committee of Shanghai Sixth People's Hospital Affiliated to Shanghai Jiao Tong University School of Medicine, and all subjects provided informed consent. All methods were carried out in accordance with relevant guidelines and regulations.

### Statistical analysis

The data were expressed as means ± standard deviation (SD), unless noted otherwise. Bioinformatics analyses were performed using R language (version 4.3.2, https://cloud.r-project.org/; R Core Team) in the RStudio environment (version 2023.09.1 + 494, https://posit.co/; RStudio Team). Statistical analysis was performed using SPSS (version 26.0, https://www.ibm.com/spss; IBM) and GraphPad Prism (version 9.0, https://www.graphpad.com/; GraphPad Software). Mann Whitney U test was used to compare the differences between the control group and LPS treated group, and *p* < 0.05 was deemed statistically significant.

## Results

### LPS intervention induces damage to HNEpC

**Figure 1:**
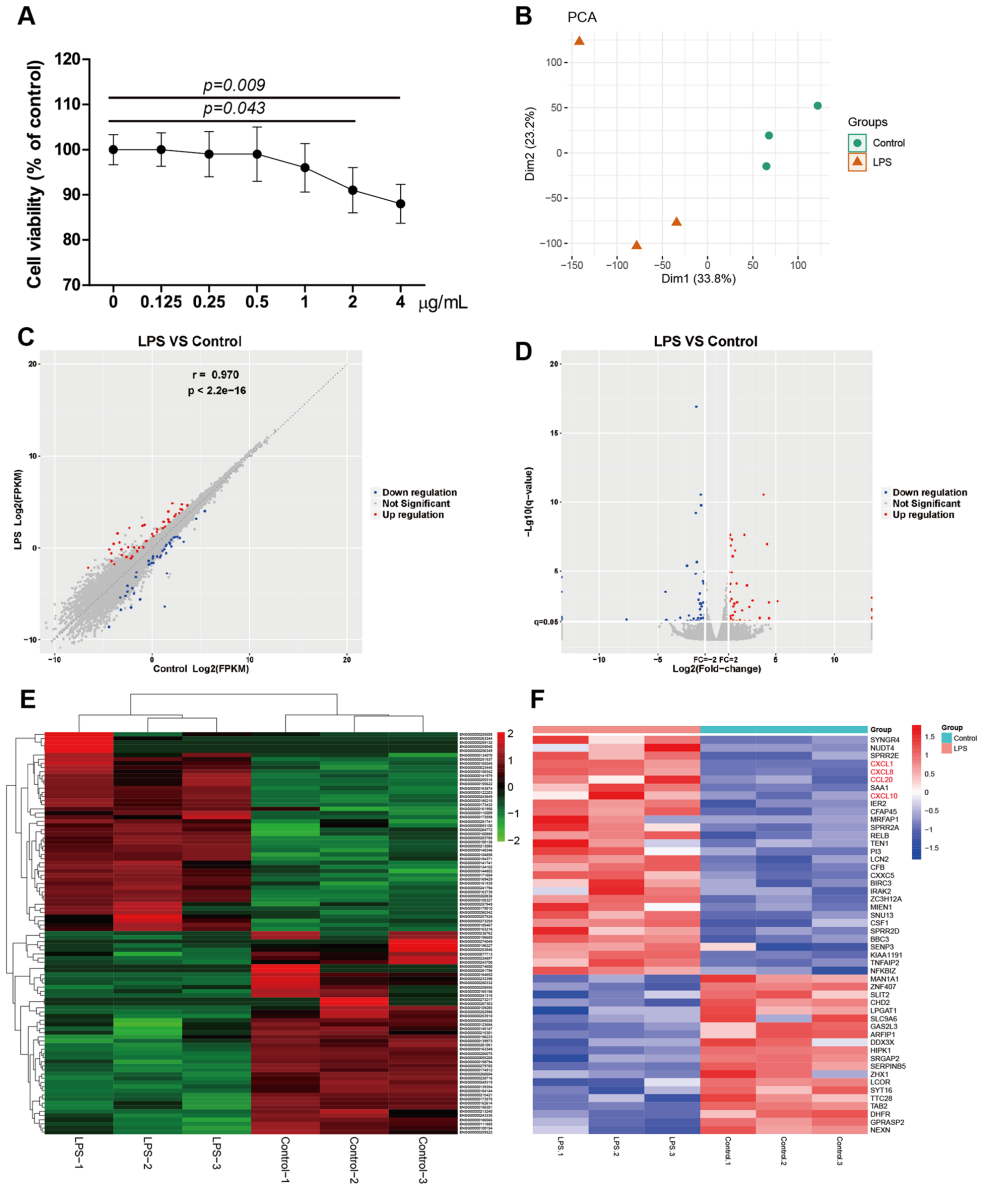
(**A**) Cell viability of HNEpC treated with LPS at concentrations of 0.125, 0.25, 0.5, 1, 2, and 4 μg/mL for 24 h. (**B**) A PCA plot revealed a distinct separation between the LPS intervention group and control group. DEGs between HNEpC treated with LPS (1 μg/mL) or not (**C**–**E**). (**C**) Correlation scatter diagram of DEGs. (**D**) Volcano plot of DEGs. (**E**) Clustered heatmap of DEGs. (**F**) Heatmap displaying the top 50 significant DEGs, including 30 upregulated DEGs and 20 downregulated DEGs.

The MTT cell survival test results illustrate a significant decrease in cell viability upon stimulation with LPS at concentrations of 2 μg/mL (*p* = 0.043) and 4 μg/mL (*p* = 0.009, Fig. 1A).

### LPS alters the transcriptome of HNEpC

The PCA plot reveals a clear differentiation between the LPS stimulation group (located on the left) and the control group (distributed on the right), highlighting the pronounced disparity between the two groups (Fig. 1B). To identify DEGs following LPS intervention, the "edgeR" R package was employed with screening criteria set at | log2fold change |> 1.0 and *q* value < 0.05. A total of 97 genes were identified as DEGs, comprising 48 up-regulated genes and 49 down-regulated genes (Fig. 1C–E). The clustered heatmap of all DEGs is presented in Fig. 1E, and the complete lists of DEGs are available in Table S2. The top 50 significant DEGs, exhibiting the highest log2 fold changes, are depicted in Fig. 1F.

### Functional enrichment analyses of DEGs

**Figure 2:**
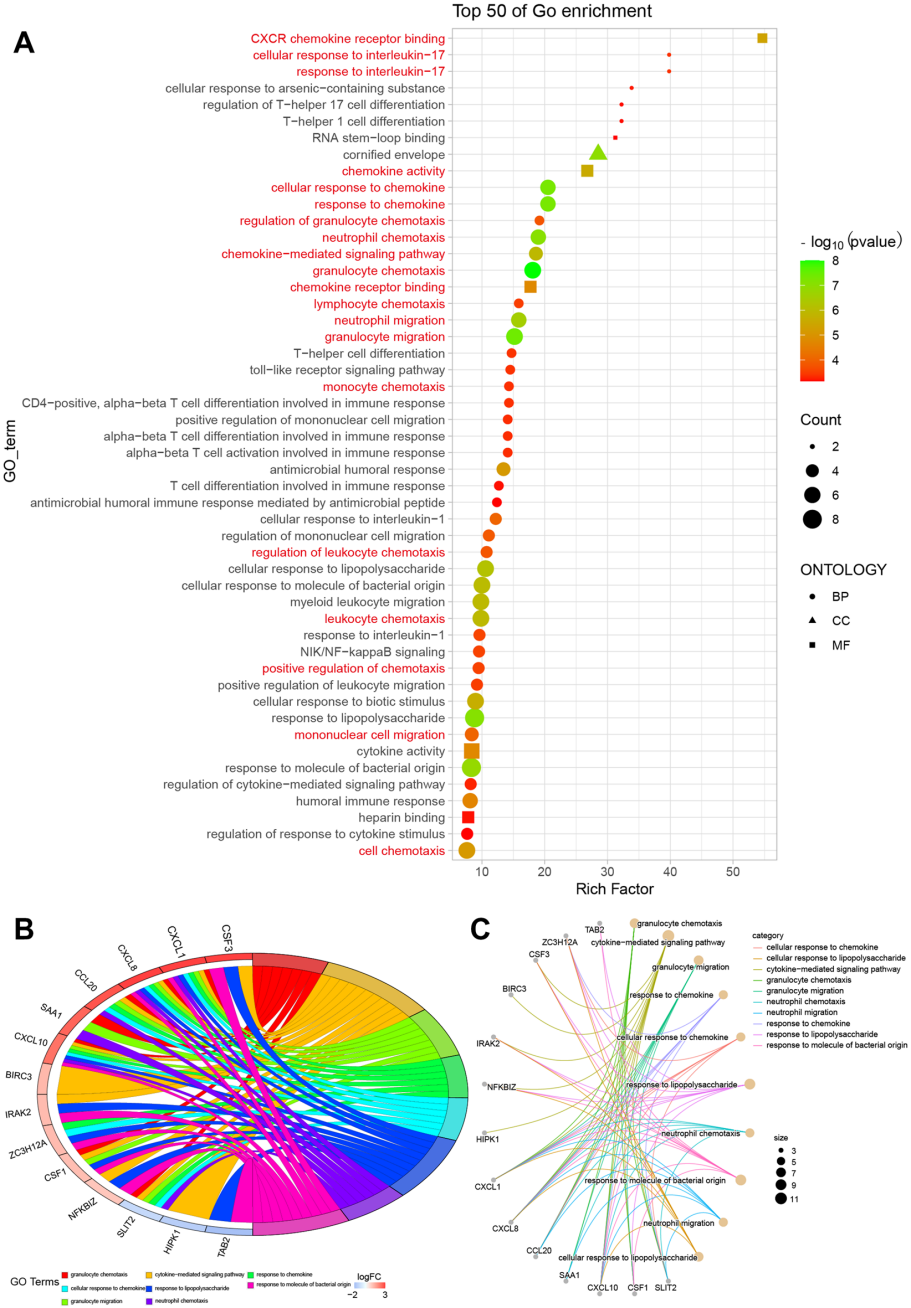
GO enrichment analysis of DEGs. (**A**) Bubble plot showing the top 50 GO terms with the highest rich factors. (**B**) Chord diagram illustrating the log2 fold changes of genes involved in selected GO terms. (**C**) Circle diagram of selected GO terms and relevant genes.

Subsequently, we conducted GO, KEGG and GSEA pathways analyses to elucidate the biological functions of DEGs. We identified 59 significantly enriched GO terms (*q* < 0.05), encompassing 50 terms in biological process (BP), 1 term in cellular component (CC), and 8 terms in molecular function (MF) (Table S3). A bubble plot displaying the top 50 GO terms with the highest rich factors is presented in Fig. 2A. Notably, chemokine-related signaling pathways, including CXCR chemokine receptor binding, cellular response to interleukin-17 (IL-17), and response to chemokine, were significantly enriched GO terms. In order to further visualize the relationship between enriched GO term or KEGG pathways and related genes, we used chord and circle diagrams for visualization. In a chord diagram, each arc in the diagram represents a GO term or KEGG pathway, and the chords connecting the arcs indicate the genes that are shared between those terms. In a circle diagram, each circle in the diagram represents a GO term or KEGG pathway. The size of the circle is proportional to the significance or enrichment level of the term or pathway, and the size of the circle is directly proportional to the number of genes associated with the GO term or KEGG pathway. Highly enriched terms or pathways have larger circles, while less enriched terms have smaller circles. Notably, the relationships between enriched GO terms and related genes are clearly and intuitively displayed (Fig. 2B,C). Enriched GO terms such as granulocyte chemotaxis, response to chemokine, cellular response to chemokine, granulocyte migration, and neutrophil chemotaxis are represented by arcs in the diagram. Key genes associated with these GO terms, including CSF3, CXCL1, CXCL8, CCL20, SAA1, CXCL10, et al., are indicated by the arcs connecting the GO term chords, or the gene circles connecting the GO term circles (Fig. 2B,C).

**Figure 3:**
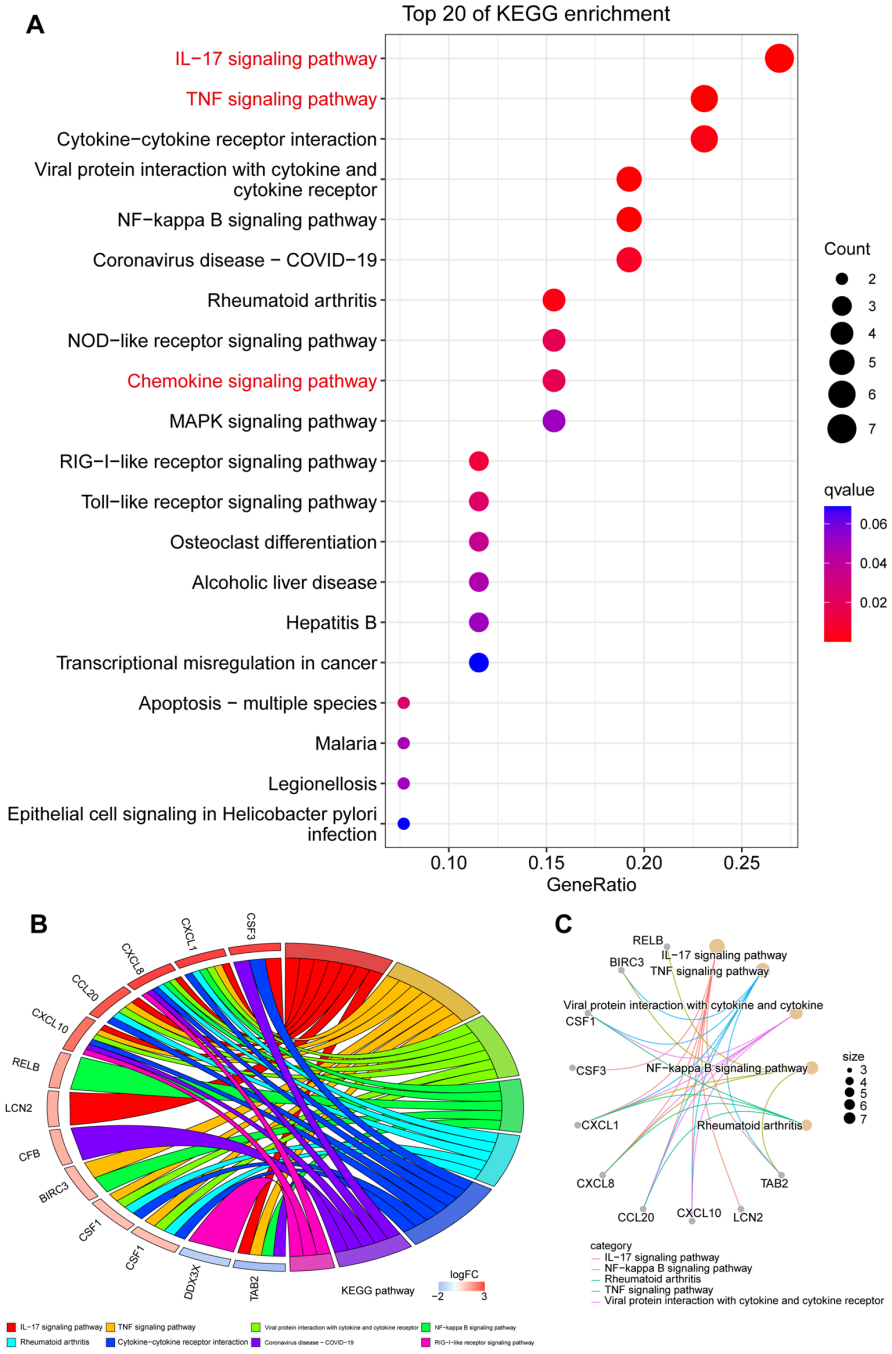
KEGG enrichment analysis of DEGs. (**A**) Bubble plot showing the top 20 KEGG pathways with the highest GeneRatio. (**B**) Chord diagram illustrating the log2 fold changes of genes involved in selected KEGG pathways. (**C**) Circle diagram of crucial KEGG pathways and relevant genes.

Next, based on KEGG database^13–15^, KEGG pathway enrichment analysis of DEGs identified 15 significantly enriched pathways (*q* < 0.05) (Table S4). The top 20 KEGG pathways with the highest GeneRatio were displayed in a bubble plot (Fig. 3A), revealing the significant enrichment of chemokine-related signaling pathways, including IL-17 signaling pathway and TNF signaling pathway. Notably, the relationships between enriched KEGG pathways and related genes are clearly and intuitively displayed (Fig. 3B,C). Enriched KEGG pathways such as IL-17 signaling pathway, TNF signaling pathway, NF-kappa B signaling pathway, et al. are represented by arcs in the diagram. Key genes associated with these KEGG pathways, including CSF3, CXCL1, CXCL8, CCL20, CXCL10, et al., are indicated by the arcs connecting the KEGG pathways chords, or the gene circles connecting the KEGG pathways circles (Fig. 3B,C).

**Figure 4:**
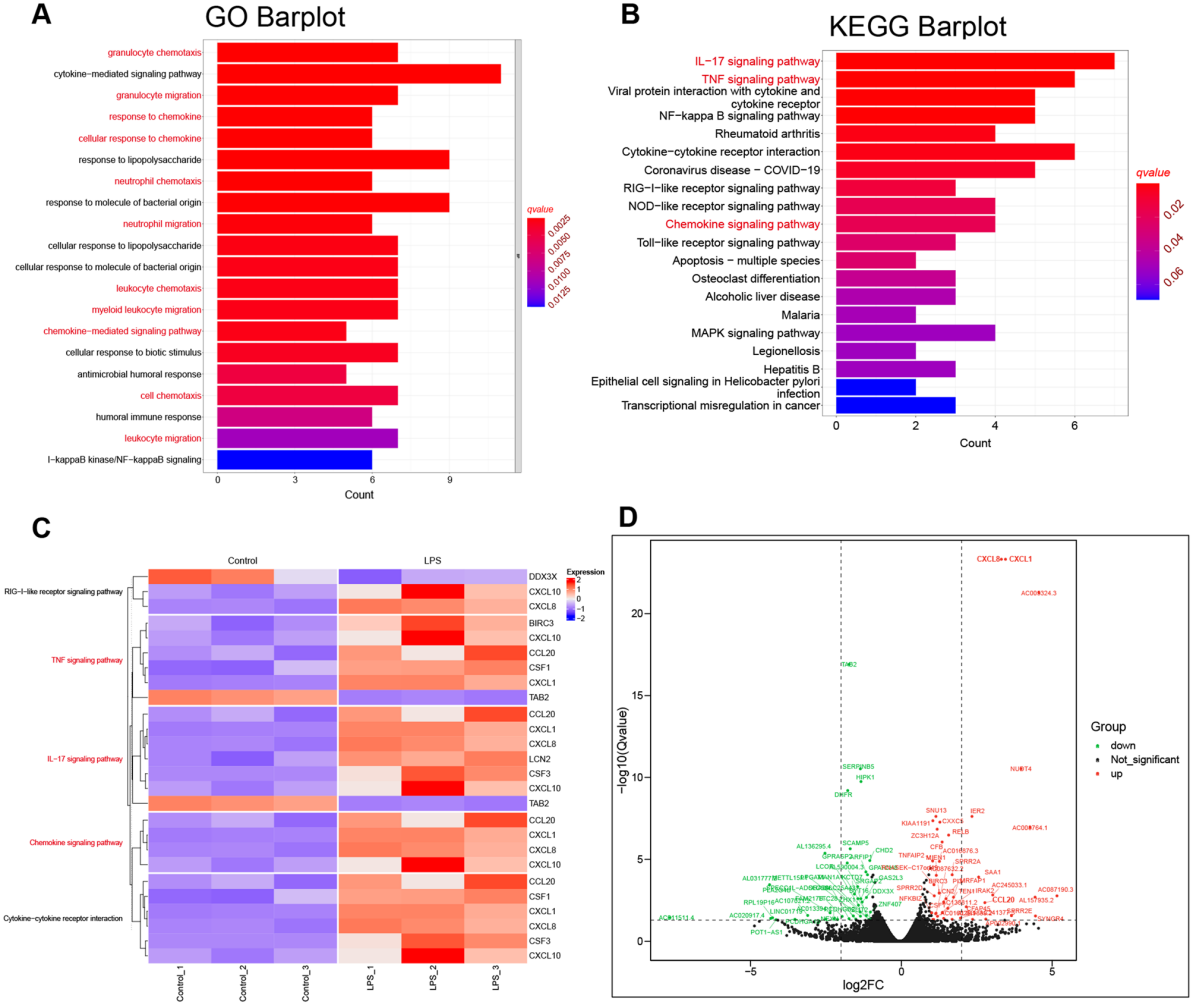
GO and KEGG enrichment analysis of DEGs. (**A**) Barplot of enriched GO pathways. (**B**) Bar plot of enriched KEGG pathways. (**C**) Heatmap of crucial KEGG pathways and the relative expression levels of relevant genes. (**D**) Volcano plot of DEGs highlighting the genes involved in the crucial enriched KEGG pathways. Genes in chemokines related signaling pathways were primarily distributed in the upper right quadrant.

The GO and KEGG enrichment analyses consistently demonstrated significant enrichment of chemokine-related signaling pathways, including CXCR chemokine receptor binding, cellular response to IL-17, chemokine activity, granulocyte chemotaxis, and cell chemotaxis pathways (Fig. 4A,B). Additionally, genes involved in chemokine signaling pathway including CXCL10, CXCL8, CXCL1 and CCL20 also participated in several other pathways including IL-17 signaling pathway, TNF signaling pathway and Cytokine-cytokine receptor interaction (Fig. 4C). Notably, volcano plot analysis highlighted key chemokines, including CXCL8, CXCL1, and CCL20, as the most significantly altered genes in LPS-treated HNEpC (Fig. 4D).

**Figure 5:**
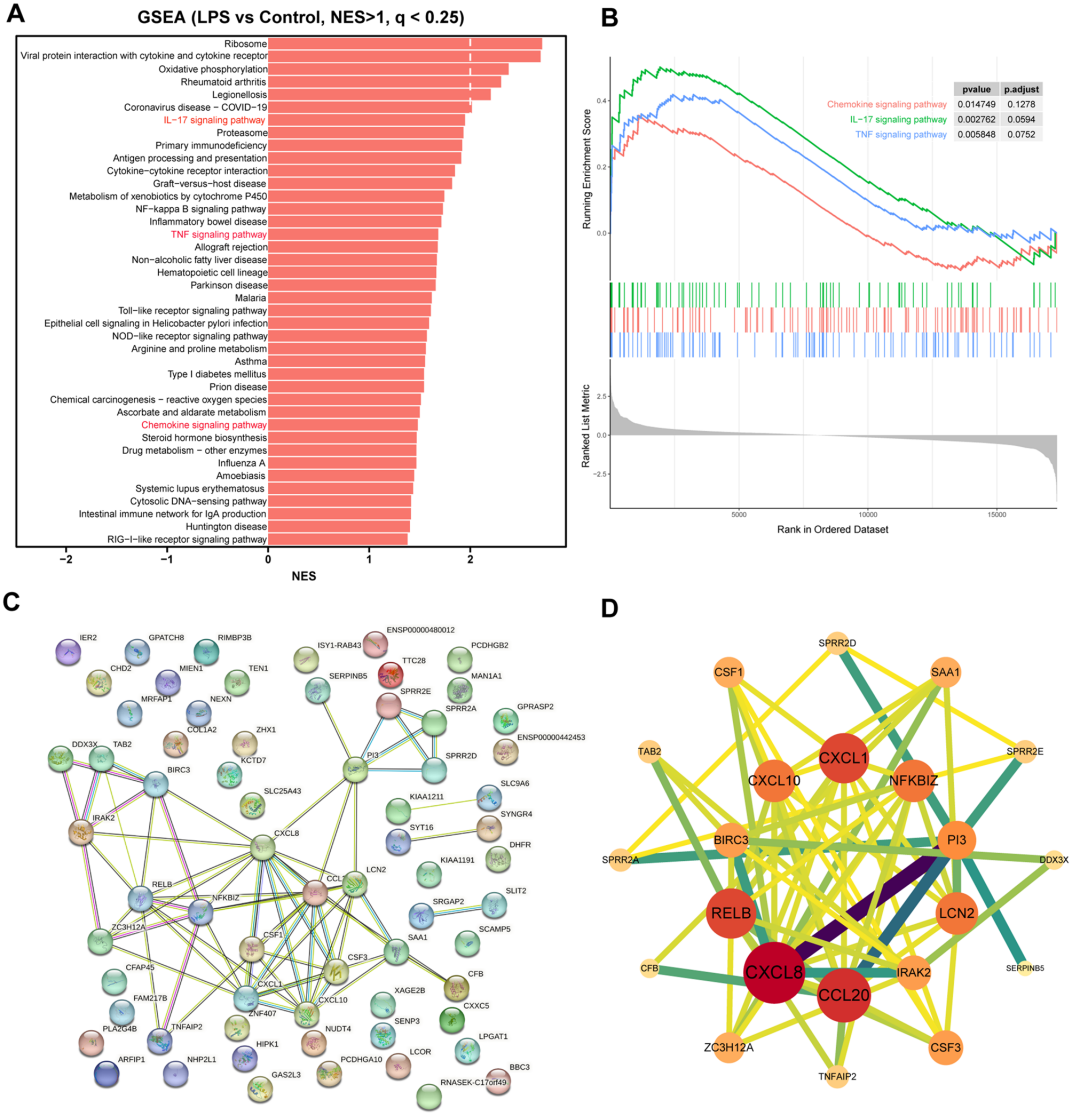
GSEA and PPI network of HNEpC treated with LPS or not. (**A**) Barplot of NES of pathways significantly enriched in HNEpC treated with LPS or not. (**B**) GSEA enrichment plot of IL-17 signaling pathway, TNF signaling pathway, and chemokine signaling pathway in HNEpC treated with LPS or not. (**C**) PPI network of DEGs constructed by Cytoscape software. (**D**) The top 22 hub genes based on the closeness parameter calculated by Cytoscape software.

To further elucidate critical pathways enriched in HNEpC treated with LPS, GSEA was conducted utilizing the KEGG database. The GSEA barplot and enrichment plot (Fig. 5A,B) revealed that, in line with the previously obtained GO and KEGG results, the chemokine signaling pathway (*p*-value = 0.014749, *p*.adjust = 0.1278), IL-17 signaling pathway (*p*-value = 0.002762, *p*.adjust = 0.0594), and TNF signaling pathway (*p*-value = 0.005848, *p*.adjust = 0.0752) exhibited significant enrichment in HNEpC treated with LPS.

### PPI network of DEGs

For the PPI network of DEGs, a network comprising 67 nodes and 67 edges was established by uploading potential targets (DEGs) from HNEpC treated with LPS to the STRING database (https://www.string-db.org/). After excluding genes with low connectivity, the resulting PPI network featured 67 nodes and 67 edges (Fig. 5C). The cytoHubba plugin identified the top 22 hub genes based on the closeness parameter, including CXCL8, CCL20, CXCL1, RELB, LCN2, CXCL10, NFKBIZ, PI3, CSF3, and IRAK2, among others. Notably, genes associated with chemokine-related signaling pathways, such as CXCL8, CCL20, CXCL1, RELB, LCN2, CXCL10, NFKBIZ, PI3, CSF3, and IRAK2, ranked among the top 10 hub genes with the highest degree values in the innermost circle (Fig. 5D).

### IPA of DEGs

**Figure 6:**
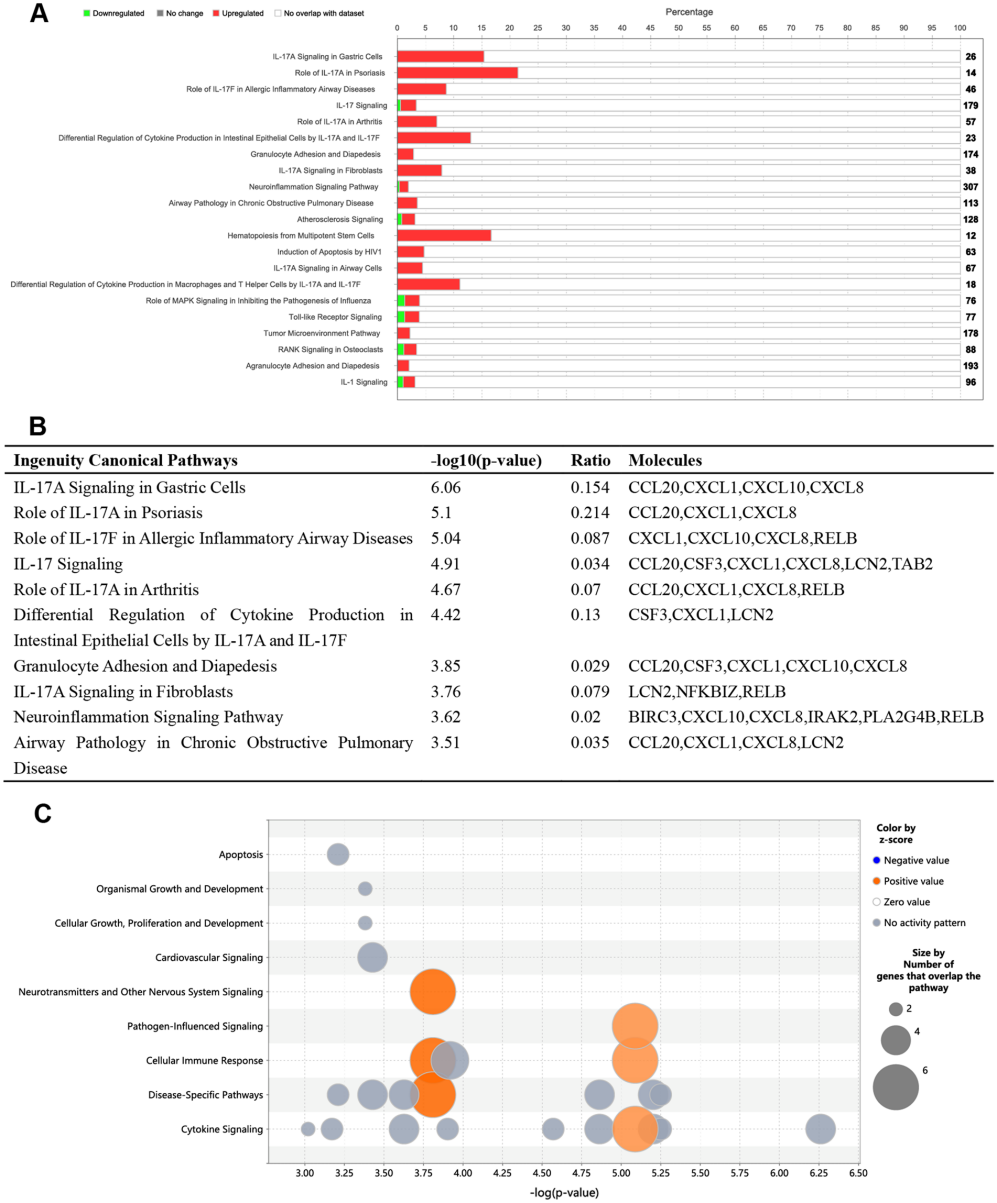
IPA of the LPS treated and control HNEpC dataset. (**A**) Histogram of the most significantly enriched ingenuity canonical pathways (− log10 (*p*-value) > 2.5). The number in the right side of the figure represents the number of all genes in this pathway, and the length of the red bar represents the percentage of upregulated (red) or downregulated (green) genes in this pathway identified through IPA analysis on the HNEpC dataset. (**B**) Top 10 ingenuity canonical pathways with − log10 (*p*-value), Ratio, and Molecules. The ratio represents the ratio of the number of molecules to the total number of molecules in this pathway. (**C**) Bubble plot indicating that neurotransmitters and other nervous system signaling, pathogen-influenced signaling, cellular immune response, disease-specific pathways, and cytokine signaling pathways were pathways with the highest *z*-score and the largest number of genes.

The IPA of DEGs involved a functional enrichment analysis using the "core analysis" function. Pathways with a –log10 (*p*-value) > 2.5 were visualized in Fig. 6A, along with corresponding *z*-scores indicating positive or negative correlation with the intervention. The IPA analysis (Fig. 6A,B) revealed a significant upregulation of IL-17A signaling-related pathways, primarily encompassing CCL20, CXCL1, CXCL0, and CXCL8 in LPS-treated HNEpC. Additionally, pathways related to neurotransmitters, nervous system signaling, pathogen-influenced signaling, cellular immune response, disease-specific pathways, and cytokine signaling pathways exhibited the highest *z*-scores and the largest number of genes (Fig. 6C).

**Figure 7:**
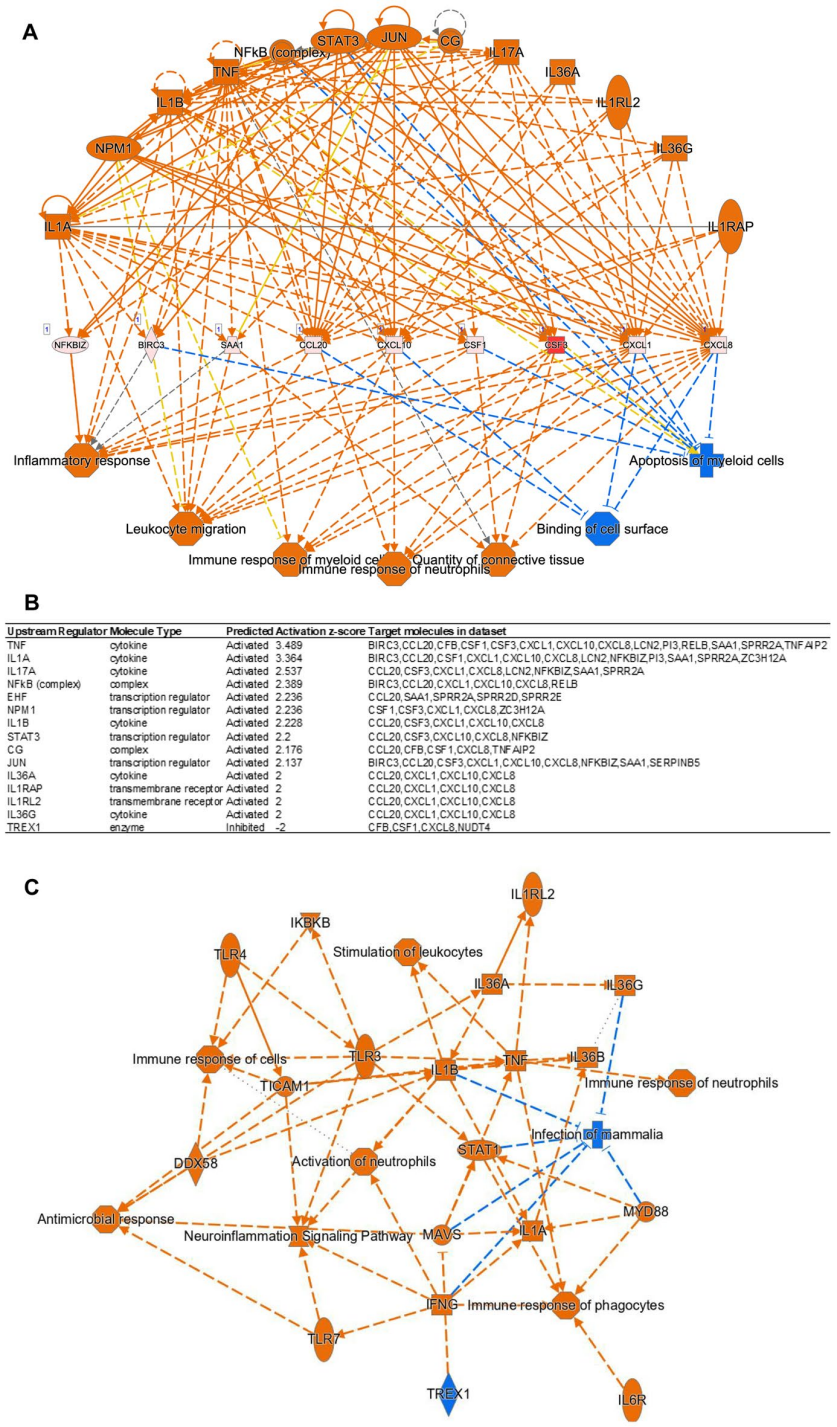
(**A**) Key upstream regulators (CG, IL17A, IL1A, IL1B, IL1RAP, IL1RL2, IL36A, IL36G, JUN, NFkB (complex), NPM1, STAT3, TNF) and target molecules (BIRC3, CCL20, CSF1, CSF3, CXCL1, CXCL10, CXCL8, NFKBIZ, SAA1) in the LPS treated and control HNEpC dataset. (**B**) Detailed information on upstream regulators in the LPS treated and control HNEpC dataset. (**C**) Graphical summary of the LPS treated and control HNEpC dataset.

Furthermore, upstream regulators and target molecules analyses (Fig. 7A,B) identified CG, IL17A, IL1A, IL1B, IL1RAP, IL1RL2, IL36A, IL36G, JUN, NFkB (complex), NPM1, STAT3, and TNF as upstream regulators, while BIRC3, CCL20, CSF1, CSF3, CXCL1, CXCL10, CXCL8, NFKBIZ, and SAA1 were identified as key target molecules in the LPS-treated and control HNEpC dataset. This suggests that key genes in chemokine-related signaling pathways are crucial target molecules in our dataset, consistent with the results of the GO and KEGG enrichment analyses.

Moreover, the graphical summary of the LPS-treated and control HNEpC dataset (Fig. 7C) illustrated that LPS induces stimulation of leukocytes, immune response of cells, antimicrobial response, neuroinflammation signaling pathway, IFNG immune response of phagocytes, immune response of neutrophils, and infection of mammalia.

### Gene expressions validation by qRT‐PCR analysis

**Figure 8:**
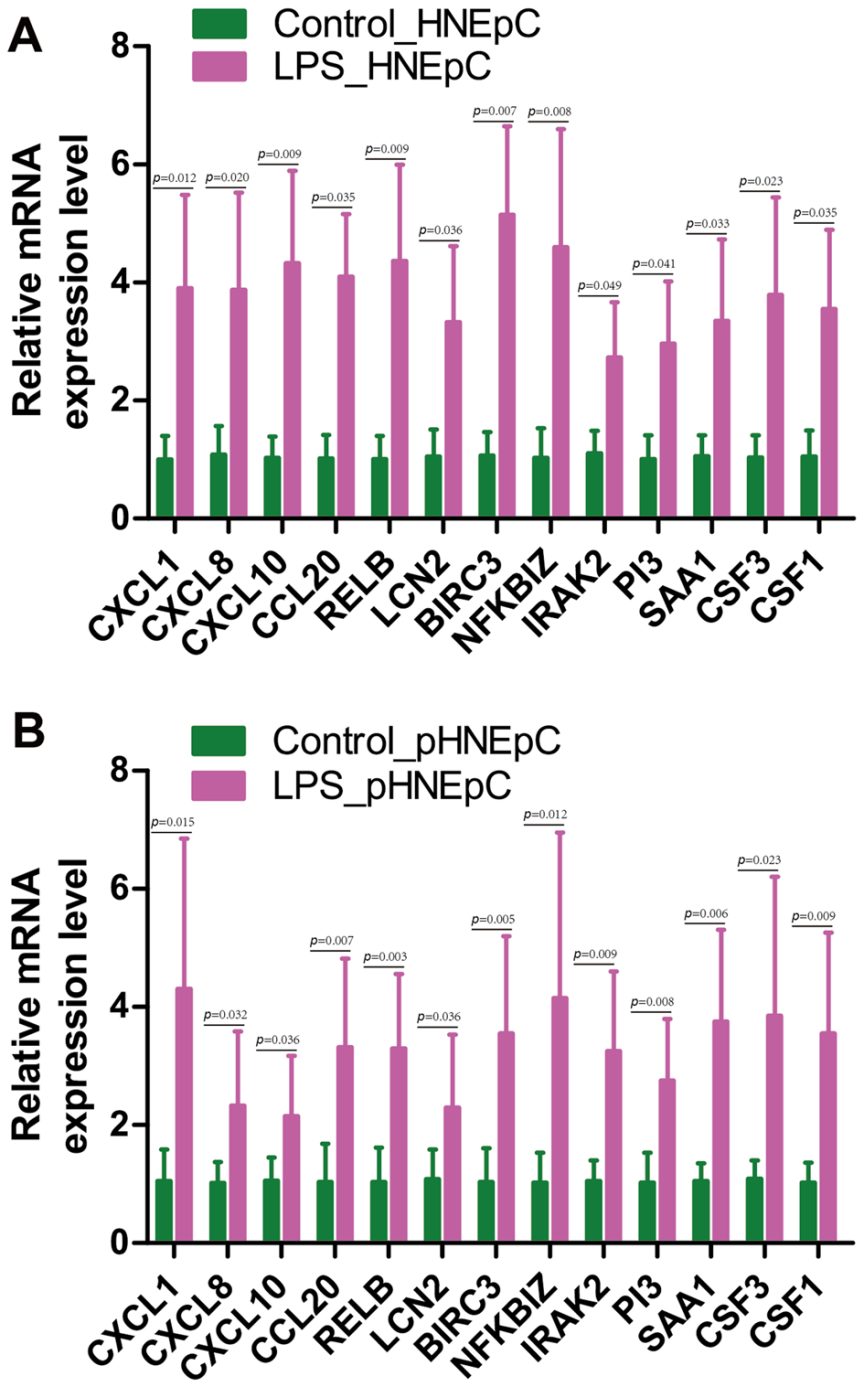
Validation of genes in chemokines related signaling pathways by qRT-PCR. The relative expression levels of CXCL1, CXCL8, CXCL10, CCL20, RELB, LCN2, BIRC3, NFKBIZ, IRAK2, PI3, SAA1, CSF3, and CSF1 in HNEpC (**A**) or pHNEpC (**B**) treated with LPS or not. GAPDH was used as a reference. Each group contained three biological sample repeats, and each sample contained three technical repeats of qRT-PCR.

To validate the gene expressions, qRT-PCR analysis was performed. The results confirmed a significant increase in the relative mRNA expression levels of CXCL1, CXCL8, CXCL10, CCL20, RELB, LCN2, BIRC3, NFKBIZ, IRAK2, PI3, SAA1, CSF3, and CSF1 in HNEpC or pHNEpC treated with LPS compared to the control group (Fig. 8A,B). Therefore, it can be concluded that chemokine-related signaling pathways may play a pivotal role in the pathogenesis of LPS-induced inflammatory responses in nasal epithelial cells.

## Discussion

HNEpC, situated on the nasal cavity's inner surface, function as a primary defense against inhaled pathogens, particles, and irritants^7,16^. Interaction between LPS and HNEpC in nasal mucosal tissues triggers immune responses and inflammatory reactions. This interaction leads to the production and release of pro-inflammatory cytokines, chemokines, and other immune mediators that signal immune cell recruitment, particularly neutrophils and macrophages, to the nasal passages. These immune cells play crucial roles in combatting infections by engulfing pathogens and contributing to the inflammatory responses^17^.

In this study, RNA sequencing and bioinformatics analyses revealed a total of 97 DEGs, with 48 up-regulated and 49 down-regulated genes between LPS-treated HNEpC and control groups (Fig. 1 and Table S2). This indicates that LPS induces transcriptome alterations in HNEpC, aligning with previous findings in porcine peripheral blood mononuclear cells^18^ and mouse macrophages^19^.

Subsequent GO, KEGG, and GSEA analyses were employed to delineate the biological functions of DEGs. A total of 59 significantly enriched GO terms (*q* < 0.05) were identified (Table S3). The top 50 GO terms, with the highest enrichment factors, were depicted in a bubble plot (Fig. 2A). Notably, chemokine-related signaling pathways, such as CXCR chemokine receptor binding, cellular response to IL-17, granulocyte chemotaxis, neutrophil migration, and granulocyte migration, were among the significantly enriched GO terms. KEGG analysis results (Table S4, Figs. 3, and 4B) further confirmed significant enrichment in chemokine-related signaling pathways, including the IL-17 signaling pathway, TNF signaling pathway, and chemokine signaling pathway. Then, we utilized chord diagrams and circle diagrams to visually and intuitively represent the relationships between enriched GO terms or KEGG pathways and the genes associated with them, and further confirmed that chemokine-related GO terms or KEGG pathways were enriched and the interconnectedness between these processes (represented by GO terms or KEGG pathways) and the genes involved were close (Figs. 2B,C, 3B,C). Genes participating in the chemokine signaling pathway, such as CXCL10, CXCL8, CXCL1, and CCL20, were also involved in related pathways, including IL-17 signaling, TNF signaling, and cytokine-cytokine receptor interaction (Fig. 4C). Additionally, GSEA analysis reinforced the significant enrichment of chemokine signaling, IL-17 signaling, and TNF signaling pathways in LPS-treated HNEpC (Fig. 5A,B). Collectively, these results indicate the activation of chemokine-related signaling pathways in response to LPS stimulation in HNEpC, crucial for recruiting inflammatory cells to combat infections and induce inflammatory responses in nasal mucosal tissues^17,20^.

In our investigation, we employed GO, KEGG, and GSEA to elucidate the biological functions of DEGs. We identified a total of 59 significantly enriched GO terms (*q* < 0.05), with the top 50 terms and their respective enrichment factors displayed in a bubble plot (Fig. 2A) and detailed in Table S3. Notably, these enriched GO terms highlighted the involvement of chemokine-related signaling pathways, such as CXCR chemokine receptor binding, cellular response to IL-17, granulocyte chemotaxis, neutrophil migration, granulocyte migration, among others. Our KEGG analysis results (Table S4, Figs. 3, and 4B) further revealed the significant enrichment of chemokine-related signaling pathways, including the IL-17 signaling pathway, TNF signaling pathway, and chemokine signaling pathway. Intriguingly, several genes participating in the chemokine signaling pathway, such as CXCL10, CXCL8, CXCL1, and CCL20, were also implicated in other pathways, including IL-17 signaling, TNF signaling, and Cytokine-cytokine receptor interaction (Fig. 4C). These key chemokines, including CXCL8, CXCL1, and CCL20, were prominently positioned in the upper right quadrant of the volcano plot, signifying their significant alteration in HNEpC treated with LPS (Fig. 4D). Subsequent GSEA analysis validated the enrichment of chemokine signaling pathway, IL-17 signaling pathway, and TNF signaling pathway in LPS-treated HNEpC (Fig. 5A,B). In summary, our comprehensive analyses demonstrate the activation of chemokine-related signaling pathways in HNEpC following LPS stimulation. This activation is crucial for recruiting inflammatory cells, such as eosinophils, neutrophils, macrophages, T cells, and B cells, to combat infections, thereby inducing an inflammatory response in nasal mucosal tissues. This phenomenon may play a pivotal role in the pathogenesis of nasal inflammation^17,20^.

Subsequently, a PPI network analysis identified genes in chemokine-related signaling pathways, including CXCL8, CCL20, CXCL1, RELB, LCN2, CXCL10, NFKBIZ, PI3, CSF3, and IRAK2, among the top 10 hub genes with the highest degree values located in the innermost circle (Fig. 5D). Further investigation using IPA revealed a significant upregulation of IL-17A signaling-related pathways, primarily involving CCL20, CXCL1, CXCL0, and CXCL8, in LPS-treated HNEpC (Fig. 6A,B). Additionally, IPA upstream regulators and target molecules analyses (Fig. 7A,B) identified BIRC3, CCL20, CSF1, CSF3, CXCL1, CXCL10, CXCL8, NFKBIZ, and SAA1 as key target molecules in both LPS-treated and control HNEpC datasets. This finding is consistent with the earlier results of GO and KEGG enrichment analyses. The graphical summary of the LPS-treated and control HNEpC dataset (Fig. 7C) illustrated that LPS induces the stimulation of leukocytes, immune cell responses, antimicrobial reactions, neuroinflammation signaling, IFNG immune responses, neutrophil immune responses, and mammalian infections. These outcomes align with previous reports indicating that LPS triggers and activates immune responses in various leukocytes, including eosinophils^21^, neutrophils^22^, macrophages^23^, T cells^24^, B cells^25^, and mast cells^26^.

**Figure 9:**
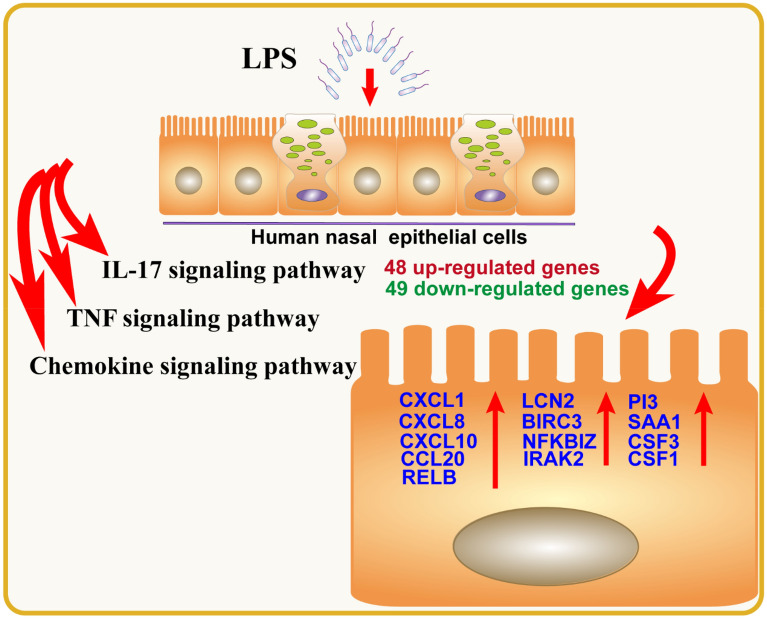
Schematic diagram displaying a hypothetical chain of events in which LPS intervention could markedly change the transcriptome of HNEpC, and then LPS could trigger inflammatory responses of HNEpC by regulating the expression of key genes in chemokines related signaling pathways.

Finally, the results of qRT-PCR confirmed a significant elevation in the relative mRNA expression levels of key genes, including CXCL1, CXCL8, CXCL10, CCL20, RELB, LCN2, BIRC3, NFKBIZ, IRAK2, PI3, SAA1, CSF3, and CSF1, in LPS-treated HNEpC or pHNEpC compared to the control group (Fig. 8). This substantiates the activation of key genes in chemokine-related signaling pathways and underscores their crucial role in the pathogenesis of LPS-induced inflammatory responses in HNEpC. As depicted in Fig. 9, the transcriptome of HNEpC undergoes significant alterations following LPS intervention. Our bioinformatics analysis identified 97 DEGs, with chemokine-related signaling pathways, including the IL-17 signaling pathway, TNF signaling pathway, and chemokine signaling pathway, prominently activated in LPS-treated HNEpC. This activation suggests that LPS regulates the expression of key genes in these signaling pathways, further contributing to inflammatory responses in HNEpC.

As stated above, HNEpC act as the first line of defense against inhaled pathogens, such as LPS, inducing inflammatory responses upon contact in nasal mucosal tissues. This interaction leads to the increased production and release of chemokines and the activation of related signaling pathways, including the IL-17 signaling pathway, TNF signaling pathway, and chemokine signaling pathway. Consequently, immune cells, including eosinophils^21^, neutrophils^22^, macrophages^23^, T cells^24^, B cells^25^, and mast cells^26^ are recruited and migrate to the site of bacterial invasion. This orchestrated immune response aims to combat infections by inducing inflammatory immune responses and engulfing pathogens^14^.

Some limitations of our study need to be discussed. First, to obtain a stable baseline of mRNA expression for RNA sequencing and avoid heterogeneity which may confuse the effects of LPS stimulation, we chose a commercial cell line HNEpC cell (PromoCell) with greater stability, less genetic variation and heterogeneity, and higher repeatability than primary epithelial cells directly isolated from human or animal tissues for cell culture and stimulation experiment. However, given the biological diversity and donor-specific effects, more primary epithelial cells directly isolated from more human or animal tissues by enzymatic or mechanical methods need to be collected to further precisely assess the impact of LPS on the transcriptome of nasal epithelial cells in the future. Second, we only used LPS originated from Escherichia coli in the present study, however, there are also other gram-negative bacteria detected in nasal cavity of chronic rhinosinusitis patients^27–29^, thus, other LPS derived from other gram-negative bacteria including Fusobacterium species, Proteus vulgaris, Salmonella typhosa, Klebsiella pneumonia and Pseudomonas aeruginosa need to be used to further comprehensively evaluate the impact of LPS on the transcriptome and inflammatory responses of nasal epithelial cells in the future.

## Conclusions

LPS intervention significantly alters the transcriptome of HNEpC. LPS may exacerbate inflammatory responses in HNEpC by activating chemokine-related signaling pathways.

### Supplementary Information

Supplementary Information 1.

Supplementary Table S1.

Supplementary Table S2.

Supplementary Table S3.

Supplementary Table S4.

Supplementary Table S5.

Supplementary Table S6.

## Data Availability

The datasets presented in this study can be found in online repositories. The names of the repository/repositories and accession number(s) can be found as below: https://www.ncbi.nlm.nih.gov/geo/, GSE241554.
