# Supplementary material for: Insights from bioinformatics analysis reveal that lipopolysaccharide induces activation of chemokine-related signaling pathways in human nasal epithelial cells
Source: Sci Rep. 2024 Apr 1;14:7672. doi: 10.1038/s41598-024-58317-y (PMC10984988; doi:10.1038/s41598-024-58317-y)
Supplement: Supplementary file 1 — Supplementary Information 1. [file 41598_2024_58317_MOESM1_ESM.docx]

**Supporting Information**

**Insights from bioinformatics analysis reveal that lipopolysaccharide induces activation of chemokine-related signaling pathways in human nasal epithelial cells**

**METHODS**

**Cell viability tests**

HNEpC (PromoCell, C-12620, Heidelberg, Germany) were cultured in an airway epithelial cell basal medium (PromoCell, C-21060) with growth medium kit (PromoCell, C- 21160) in a 5% CO_2_ incubator at 37℃. HNEpC were diluted to 1×10^5^/mL with airway epithelial cell culture medium, and then seeded in sterile 96-well culture plates in a 5% CO_2_ incubator at 37℃ for 24h. Then, LPS stock solution was diluted into different concentrations including 0.125, 0.25, 0.5, 1, 2 and 4μg/mL with airway epithelial cell culture medium. The control wells were added with equal volume of culture medium and there were no cells cultured in the blank wells. Following 6 hours stimulation, 100 μl sterile PBS solution and 10 μL 1 mg/mL MTT solution (Beyotime, Shanghai, China) were added to each well, and then incubated for 4 h. Afterwards, the supernatants were sucked and discarded, and each well was washed three times. Then, 100 μL DMSO was added into each well, and then oscillated on a shaker for 10 minutes to mix well. The absorbance was measured at 570 nm using Synergy H1 multi-mode reader (Bio Tek). Cell viability = (LPS treated well - blank well)/(control well - blank well)×100%.

**Quantitative real-time reverse transcriptions PCR**

To verify the result of RNA-seq, key DEGs were chosen for qRT-PCR. Total RNA in the cells was extracted utilizing RNeasy commercial kit (Qiagen, Chatsworth, CA, USA). The purity and integrity were assessed by measuring absorbance ratios at 260/280nm (1.8~2.0 was considered eligible) and agarose gel electrophoresis, respectively. Total RNA was reverse-transcribed to cDNA and quantitative real-time PCR was conducted using SYBR Premix Ex Taq kit (TaKaRa Biotechnology, Dalian, China) with specific primers (Table S6). Incubation conditions of PCR were as follows: 95°C/30 sec, 40 cycles of 95°C/10 sec, annealing at 60°C/60 sec and extension at 72°C/20 sec. Each sample was analyzed in triplicate. Glyceraldehyde 3-phosphate dehydrogenase (GAPDH) was used as an internal control gene. Relative mRNA levels were expressed and calculated using 2(-Delta Delta CT) methods. A normal control cell sample was designated as the calibrator.

**Legends for supplementary Table 1-6**

**Table S1. Data preprocessing and genome comparing results**

**Table S2. DEGs in LPS treated and control HNEpC**

**Table S3. GO functional enrichment analyses of DEGs**

**Table S4. KEGG functional enrichment analyses of DEGs**

**Table S5. Key genes in chemokines related signaling pathways**

**Table S6. Primers used for qPCR analysis**
